# Optical and Electrical Characterization of Biocompatible Polymeric Lines for Hemodialysis Applications

**DOI:** 10.3390/ma11030438

**Published:** 2018-03-16

**Authors:** Enrico Ravagli, Stefano Severi

**Affiliations:** 1 Department of Electrical, Electronic and Information Engineering “Guglielmo Marconi”, University of Bologna, 47521 Cesena, Italy; enrico.ravagli4@unibo.it; 2 Health Sciences and Technologies-Interdepartmental Center for Industrial Research, University of Bologna, 47521 Cesena, Italy

**Keywords:** hemodialysis, biopolymer, bloodline, electrical impedance, optical absorbance

## Abstract

During hemodialysis (HD), blood is circulated through an extracorporeal tubing system (bloodline) made of medical-grade polymeric material. Sensors of various types that do not come into contact with blood (optical, electromagnetic, etc.) are applied directly across the bloodline for clinical purposes and for therapy customization. Thus, a detailed knowledge of the bloodline’s physical properties is useful for the development of next-generation HD sensors. In this work, we performed a novel comparative analysis of the materials used by the manufacturers of the bloodlines. We focused on signals and characterization techniques matching those of the abovementioned sensors; consequently, this is an application-specific study of the optical and electrical characterization of bloodline material. Such properties are analyzed and compared for bloodlines from seven different manufacturers by optical absorbance spectroscopy and electrical impedance spectroscopy (EIS). Absorbance spectrum measurements are carried out in the VIS-NIR range. Absorbance spectra are pre-processed and data from both types of analyses are normalized with respect to sample thickness. Optical analysis shows that all bloodlines except one have similarly shaped spectra with slight quantitative differences. In all optical spectra, we find a decreasing trend of specific absorption from 0.14 mm^−1^ at 400 nm to 0.06 mm^−1^ at 1000 nm, with an absorption peak at 915 nm. In one case, a large absorption peak centered at ≃600 nm is found. Electrical analysis shows that all bloodlines have the electrical properties of a constant-phase element (CPE), with statistically significant differences in parameters’ values. Estimation of electrical CPE parameters for all bloodline returns a range of 0.942–0.957 for parameter n and a range of 12.41–16.64 for parameter Q_0_’. In conclusion, we find that, although some statistically significant differences are present, bloodlines from a representative group of manufacturers share similar electrical and optical properties. Therefore, contactless sensing devices developed for HD will work on different bloodlines if a simple recalibration is performed.

## 1. Introduction

Hemodialysis (HD) is a blood purification therapy developed as a partial surrogate for kidney activity in nephropathic patients—that is, patients with low or no residual renal function [1,2].

HD is performed through apposite machines. Distinct types of machines exist to treat patients with acute kidney injury (AKI) (caused, for example, by poisoning), and patients with chronic kidney disease (CKD), who have no chance to recover renal function without a transplant. Whereas AKI is usually an emergency one-time treatment performed until renal function is recovered, CKD requires ongoing, periodic treatment. For example, on average a CKD patient will receive four-hour-long treatments three times a week. This study focuses on analyzing some specific properties of the disposable material involved in treatment of CKD patients by chronic HD. 

Several types of chronic HD therapies exist (standard hemodialysis, hemofiltration, hemodiafiltration, etc.); however, a detailed description of all variants is out of the scope of this work. Briefly, in standard HD, the blood of the patient flows through an extracorporeal circuit and across a specialized filter, the hemodialyzer. At the filter, blood is purified by contact with a sterile solution, the dialysate, flowing in counter-current on the other side of the membrane of the filter. By means of the physical mechanisms of diffusion and convection [3,4], three effects are achieved:

- Uremic toxins produced by the organism are transferred from blood to dialysate.
- Excess liquid accumulated by the patient due to lack of excretion through the kidneys is removed.
- Hematic concentrations of electrolytes are re-balanced.

**Figure 1:**
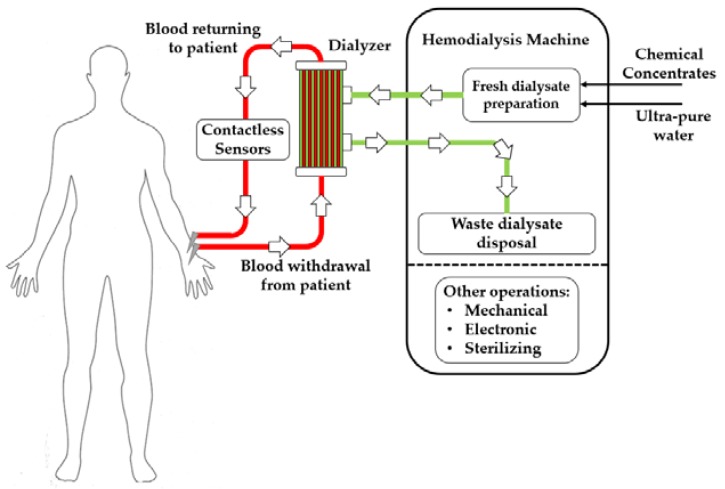
Conceptual diagram of the HD treatment for CKD patients. Red line shows the blood-side hydraulic circuit, green line the dialysate-side circuit.

Afterwards, the purified blood flows back into the patient, whereas waste dialysate is disposed of. Figure 1 shows a simplified diagram of the HD process. 

There are two hydraulic circuits, blood-side and dialysate-side; the dialysate side (green line in Figure 1) is embedded in the machine and sterilized at the end of each treatment. On the other side, the blood-side circuit (red line in Figure 1) is made of disposable material. The hemo-compatible tubing which makes up the disposable blood-side circuit is also called a bloodline. Before the start of a treatment, healthcare operators assemble the disposable kits containing bloodline, filter, and needles and connect the circuit to the machine and to the patient. 

The most widely used material for bloodlines is polyvinylchloride (PVC), with the addition of plasticizers to improve flexibility [5,6,7], but the specific composition of the tubing differs among manufacturers. Silicone is sometime used for the segment of the bloodline that goes through the roller pump, for mechanical reasons [5]. In the past, the most common type of plasticizer was Diethylhexylphthalate (DEHP). However, several investigations reported the release of plasticizer material from bloodlines into the bloodstream [6,7,8,9], and concerns regarding exposure to phthalates have risen in recent years, due to possible hepatotoxic and endocrine disruption effects [10]. Currently, DEHP is classified in Europe as toxic to reproduction, according to regulation EC 1272/2008 [11]. Thus, many manufacturing companies are now offering DEHP-free solutions for extracorporeal tubing (e.g., [12,13]). Sterilization of the disposable kits during packaging may be performed by different methods: heat, steam, ethylene oxide (EtO), or radiation (beta or gamma rays) [5,14].

Throughout the years, HD machines have been the target of technological improvements, including the use of sensors to monitor and control the treatment’s progression. Modern HD machines include many types of sensors placed in various positions, for example:

- Pressure sensors on the arterial and venous sides of the bloodline
- Pressure sensors on both sides of the hemodialyzers to monitor transmembrane pressure
- Conductivity sensors on the inlet (fresh) and outlet (waste) sides of the dialysate circuit to monitor dialysate preparation
- Optical or ultrasonic sensors to monitor the potentially dangerous formation of bubbles in the bloodline

Sensors can also be used for purposes not related to operation of the machine, such as collection of physiological data and treatment tuning. For example, hematocrit or hemoglobin sensors, which measure the optical absorbance of the blood directly in the bloodline [15,16], can be used to tune the rate of liquid removal from the blood to avoid side effects to the patient. This real-time tuning process and other similar sensor-based therapy adjustments are called biofeedback processes [17,18,19,20,21,22].

In recent years, research activity has focused on finding additional ways to extract useful information non-invasively from the HD machine—for example, by performing signal processing on pressure data to detect heart signals [23,24,25], or analyzing the optical properties of waste dialysate to detect hemolysis promptly [26]. Recently, our group developed an electrical impedance sensor able to detect changes in the conductivity of liquids inside HD bloodlines [27]. The sensor measures conductivity variation directly in the bloodline and can provide continuous real-time data. Some on-patient solutions for data collection and danger warning have also been proposed, such as the use of photopletismography (PPG) during dialysis [28,29] or the use of optical and electrical sensing to detect venous needle dislodgment [30,31]. However, these solutions have the downside of increasing the encumbrance on the patient during treatment, reducing mobility and increasing discomfort.

Contactless sensing performed directly on the blood side, intended as sensing with methods which do not require direct probe contact with blood, provides the best opportunity to collect relevant physiological data; developing next-generation HD sensors to accomplish this depends on knowing the physical properties of the bloodlines and how these properties might vary among the most common brands. 

The aim of this work is the characterization of materials used by manufacturers of the bloodlines, for use in design and calibration of optical and impedance contactless sensors for HD. We compared the electrical and optical properties of bloodline polymers from different manufacturers to understand if they are significantly different. To the best of our knowledge, this kind of comparison is novel in the literature and has never been reported.

This work has merit because some contactless sensing techniques may not be accurate or even viable on some bloodlines, depending on their properties, so a comparison is necessary. We compared bloodlines from many different companies by subjecting their disposable kits to optical absorbance spectroscopy and electrical impedance spectroscopy (EIS) [32]. Afterwards, the optical and electrical spectra of the bloodlines were compared to check for both qualitative and quantitative differences. 

It should be clearly stated that, although this study reports data on the physical properties that are related to the nature of the polymeric material of the bloodlines, the properties were directly analyzed without any consideration of the specific composition of each bloodline. We focused on signals and characterization techniques matching those of the abovementioned sensors; consequently, this is an application-specific study of the characterization of bloodline material.

## 2. Materials and Methods 

In this work, seven distinct brands of disposable hemodialysis bloodlines were analyzed by optical and electrical spectroscopy. Section 2.1 describes the different disposable kits analyzed. Section 2.2 and Section 2.3 explain the optical and electrical measurement procedures, respectively. Data from the measurements is available online.

### 2.1. List of Bloodlines and Basic Properties

For each of the seven manufacturers considered in this study, a disposable bloodline kit was collected from a local hospital or hemodialysis company. Table 1 reports the identification information from the packaging labels. Whenever some information about composition was present on the package, it is also reported in Table 1, third column. For each manufacturer, an abbreviation that will hereafter be used is indicated in the fourth column of the table.

After the kits were unpacked, the thickness and internal diameter of each bloodline were measured with an analog caliper (resolution: 0.05 mm). For each parameter, three measurements were performed and averaged. Thickness was measured because it influences the results of the optical and electrical spectroscopies. Diameter and thickness are also reported in Table 1.

### 2.2. Optical Absorbance Spectroscopy

An Infinite M200 Plate Reader (Tecan Italia Srl, Cernusco Sul Naviglio, Italy) was used to perform optical absorbance scans on samples prepared from each bloodline. This instrument, mostly used for absorbance and fluorescence studies on biological material, was available in our lab and was chosen for its capability of performing generic absorbance measurements on many samples at multiple wavelengths in an automated fashion.

It was necessary to prepare the samples, so they fit the multi-well plate of the instrument. A short segment from each bloodline was cut along its longitudinal axis and placed under mechanical pressure for a short amount of time (≃1 h) to flatten it for further mechanical manipulation. Subsequently, a dedicated mechanical tool was used to cut small disks (the same diameter as the plate’s wells) from the flattened polymer. For each brand, 20 samples were prepared, to average out possible differences in manufacturing of the samples. 

**Figure 2:**
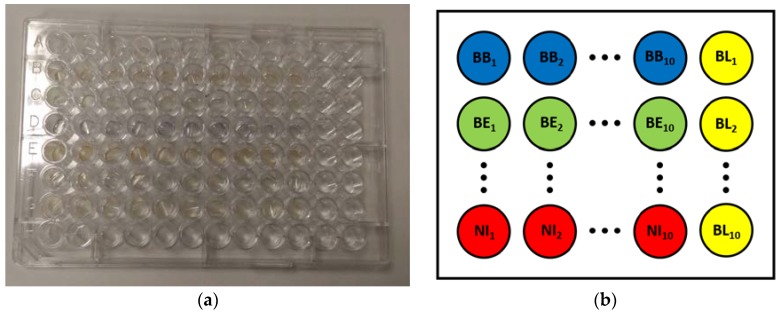
Image and diagram of the samples for optical measurement: (**a**) image of the first plate containing the bloodline samples for optical absorbance scans; and (**b**) diagram explaining the placement of samples on each plate. Each row contains ten samples from the same brand of bloodline and an empty well to serve as baseline (BL). In the second plate, the order of the rows is reversed.

Optical absorbance measurements were performed using the automatic scan procedure of the instrument. The wavelength range chosen for the measurements was 400–1000 nm, corresponding to the visible (VIS) region and part of the near-infrared (NIR) region of the spectrum. The chosen range was sampled with a resolution of 2 nm. The 20 samples for each bloodline were divided into two groups of ten and placed at different positions on two different plates. With this procedure, samples from the same bloodline were analyzed at different times during the scan, thus averaging possible thermal effects from the ongoing activity of the instrument. For each group of ten bloodline samples, a measurement on an adjacent empty well was also performed to act as a baseline. Thus, a total of 154 measurements were performed. The placement of samples on one plate is shown in Figure 2. 

Each optical spectrum obtained from a sample was pre-processed by subtracting its corresponding baseline (from the same row). The group of 20 spectra for each brand of bloodline was then averaged to obtain a mean spectrum. Absorbance (A) was measured by the instrument according to Equation (1), where I_0_ and I are the emitted and received light power at wavelength λ, respectively. Thus, they are dimensionless parameters. If the polymeric material is assumed to have a specific absorbance ratio of α(λ), expressed in 1/mm, Equation (1) can be combined with Lambert-Beer’s law in Equation (2), showing that A is the product of α and d, where d is the optical path of the measurement expressed in [mm]. This is reported in Equation (3). Thus, the specific absorbance α(λ) can be computed by normalizing A(λ) with respect to d, as shown in Equation (4). Assuming absorbance in air is negligible, d corresponds to the thickness of the bloodline, measured in Section 2.1, and thus of the sample. (In the Results Section, mean spectra for each bloodline are reported both as A(λ) and α(λ) to show both the original data and thickness-normalized data.)

(1) $$A\left(\lambda \right)=\log_{10}\frac{I_{0}\left(\lambda \right)}{I\left(\lambda \right)}$$

(2) $$I_{0}\left(\lambda \right)=I\left(\lambda \right)\cdot 10^{-\alpha \left(\lambda \right)d}$$

(3) A(λ)=α(λ)·d

(4) $$\alpha \left(\lambda \right)=\frac{A\left(\lambda \right)}{d}$$

### 2.3. Electrical Impedance Spectroscopy

**Figure 3:**
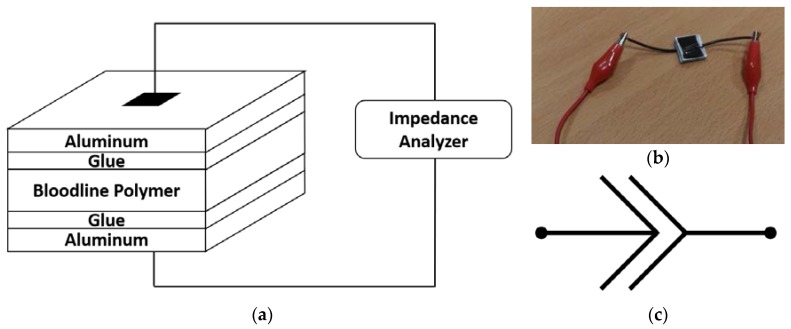
Details of the electrical analysis of the samples: (**a**) simplified diagram of the setup (not to scale); (**b**) one of the manufactured capacitor-like samples connected to the LCR meter; and (**c**) circuital constant-phase element (CPE) symbol.

An AgilentE4980A Precision LCR Meter (Agilent Technologies, Santa Clara, CA, USA) was used to perform EIS analysis. To analyze the electrical properties of each bloodline in a comparable manner, material samples were prepared in a capacitor-like manner, with a square shape of equal surface. A diagram of the measurement setup is shown in Figure 3a.

As with the optical analysis, a short segment of each type of bloodline was removed from the disposable kit and cut along its longitudinal axis, and segments were mechanically pressed to obtain flat polymeric areas. For this analysis, square samples (15 mm × 15 mm) were cut. Each polymeric sample was sandwiched between two aluminum squares of the same area (15 mm × 15 mm, with 0.8 mm thickness). The sample was fixed to the aluminum using commercial superglue on both sides. All sample-manufacturing operations were performed manually with the best possible care, to ensure an even distribution of glue across the sample surface and as much homogeneity as possible between samples. A sample, connected to the nodes of the LCR meter, is shown in Figure 3b. For each type of bloodline, five samples were prepared, for a total of 35 samples.

Before performing the measurements, the instrument was calibrated in open- and short-circuit configuration. Placement of instrument, cables and connectors has not been altered between calibration and measurements. For each sample, multiple EIS two-point measurements were taken with the LCR Meter in R-X mode (recording the real (R) and imaginary (X) parts of electrical impedance). A voltage stimulation amplitude of 1 V was used. Each measurement corresponds to a set of data points taken at 201 equally-spaced frequencies in the 1–2 MHz range. The range for the frequency sweeps was chosen by the following criteria:

- The upper bound of the instrument range was 2 MHz (20 Hz–2 MHz).
- Polymers are usually dielectric in nature, so a reasonable lower bound is required to obtain proper readings (impedance → ∞ for frequency → 0).
- Previous work by our group [27] showed that: (a) at least one bloodline type showed nearly-capacitive behavior; and (b) 1–2 MHz is a valid range choice to decrease coupling impedance and thus increase sensitivity.

For some samples, an issue was present in which recorded data was scattered all across the complex impedance plane in a configuration without any physical meaning. Whereas impedance spectra show frequency-dependent features depending on the composition of the sample under analysis, such changes usually happen with a slow transition over a group of adjacent frequencies, which was not our case. Consecutive repeated measures on the same sample showed this issue only in some iterations, whereas the others returned physically sound EIS data. We attributed this issue to external interference and discarded the noisy measurements. A second source of discarded measurements was the presence of outliers, i.e., EIS measurements much different from the other results obtained for the same sample. A possible explanation for these data, which were a large minority (eight measurements), was an unstable component of the experimental setup. The qualitative nature of the EIS analysis results (i.e. the CPE effect described here below) was also verified with an independent custom-built impedance analyzer device.

At least one valid spectroscopy result was available for each sample. A total of 134 spectroscopies were analyzed. Where multiple frequency sweeps were available, a mean impedance spectrum was computed by averaging all available spectra.

To compare EIS data using quantitative parameters, impedance spectra were fit with a lumped-parameter model using the software ZView (Scribner Associates, Inc., Southern Pines, NC, USA). After preliminary analysis of the raw EIS data and based on our previous findings [27], the constant-phase element (CPE) was chosen for fitting. The CPE [33,34,35,36] is best described as a circuital representation for an imperfect electrical capacitor. Its electrical symbol is shown in Figure 3c. Its mathematical formulation, reported in Equation (5), was created to model real-world systems which deviate from their expected capacitive behavior because they have both an active and a reactive impedance changing with frequency.

(5) $$Z_{\mathrm{CPE}}\;=\;\frac{1}{Q_{0}{\left(\mathrm{jw}\right)}^{n}}$$

Equation (5) shows that CPE impedance has indeed a constant phase angle of −n∙π/2 radians. One possible explanation for the CPE effect is the presence of inhomogeneity in the material, resulting in a continuous distribution of time constants, also called “frequency dispersion of capacitance” [35]. 

For each brand, parameters Q_0_ and n were computed, starting from the mean impedance spectra available for each of the three physical samples. Thus, for each brand, three sets [Q_0_, n] were obtained. The value of n is only dependent on the nature of the material. Thus, it was directly analyzed. However, parameter Q_0_ may also be influenced by the thickness of each sample. Thus, normalized parameter Q_0_’ was computed as shown in Equation (6), where parameter d is the thickness of the sample. Formulation of Q_0_’ was chosen assuming that modulus Q_0_ has an inverse relationship with thickness, as in perfect capacitors. Thus, using Equation (6), thickness dependence of Q_0_ is compensated.

(6) $$Q_{0}{}^{\prime }\;={\;Q}_{0}\cdot d$$

Statistical analysis of n and Q_0_’ was performed using Matlab (Mathworks, Natick, MA, USA). Parameters were subjected to one-way analysis of variance (ANOVA) test using Matlab’s statistical toolbox.

## 3. Results

### 3.1. Optical Absorbance Spectroscopy

**Figure 4:**
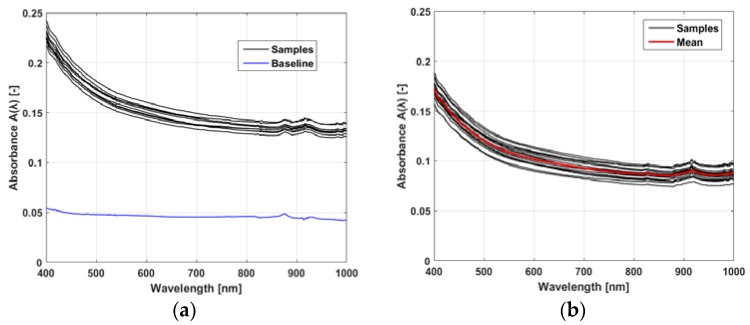
Example of raw and processed absorbance spectra A(λ) for one of bloodline brands (BG): (**a**) group of 10 spectra from a single plate (black) with their baseline (blue); and (**b**) spectra from all of the 20 samples after baseline removal (black) and their mean (red).

**Figure 5:**
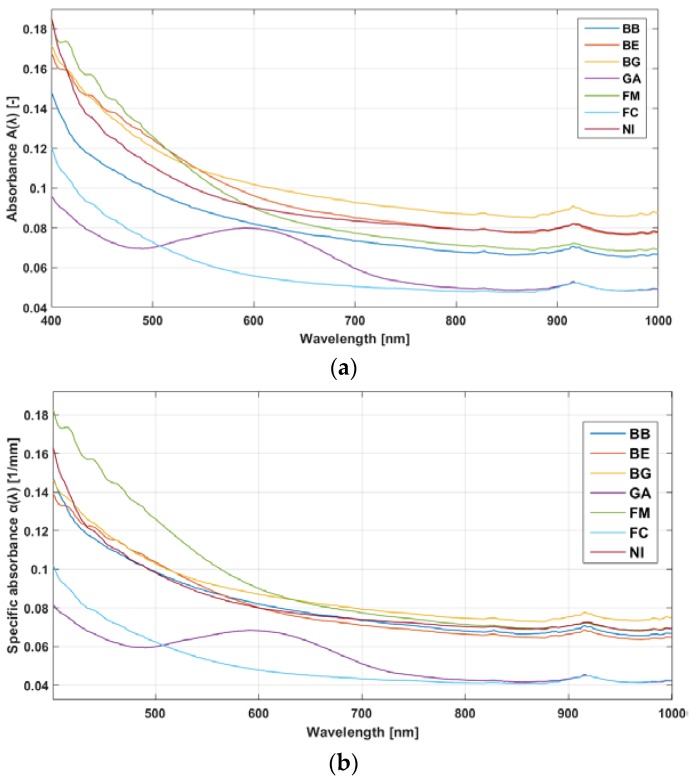
(**a**) Mean absorbance A(λ); and (**b**) mean specific absorbance α(λ) for each bloodline.

Figure 4 shows the absorbance measurements for the samples from the BG bloodline. This result is taken as an example, to show the general shape and variability of the spectra; the group of samples is representative of most of the bloodline brands, with one exception (shown in Figure 4a and Figure 5) shows that, despite some natural variability in the measurements due to slight differences in the physical preparation of the samples, all absorbance spectra (black lines) follow the same trend and are closely grouped. Figure 4a also reports the baseline trend for the empty plate (blue line). It must be emphasized that raw spectra present an exponential-like decay at the lower wavelengths and two absorbance peaks at ≃880 and 915 nm. However, the peak at 880 nm is mostly an effect of the plate absorbance, since in Figure 4b, which shows the spectra after baseline removal, there is only one significant peak in absorbance, at 915 nm. The baseline also shows a slight exponential decay, but after baseline removal the spectra of the samples still show a strong exponential decay, indicating that this behavior is mostly a property of the polymeric material of the bloodline. Figure 4b also shows the mean spectrum (red line) obtained after averaging the group of measured spectra at each wavelength. Standard deviation of baseline-free spectra was also computed for each group of samples from the same bloodline and normalized to the mean spectra of the group. A value of 8.2% standard deviation was found, averaged across all bloodlines and wavelength.

In Figure 5, raw absorbance spectra A(λ) and specific absorbance spectra α(λ) for all bloodlines are reported. As shown in the figure, the transition from A(λ) to α(λ) has the effect of bundling together a few of the spectra (BE, BG, BB, and NI). This result may be an indication of similar materials, but it may also be that different compositions have similar overall spectra. Another spectrum, from the FM samples, has specific absorbance similar to that group in the 600–1000 nm range but a different decay in the 400–600 nm range. Two spectra, from the FC and GA brands, show the most significant differences from the other brands. Both show a lower mean α(λ) level compared to the other brands. However, whereas the FC spectrum has a shape similar to the previous samples, the GA spectrum also shows an increased α(λ) in the 500–700 nm region, superimposed on the exponential-like decay. Overall, all the brands share the features of exponential-like decay of absorbance and an absorbance peak at 915 nm.

### 3.2. Electrical Impedance Spectroscopy

**Figure 6:**
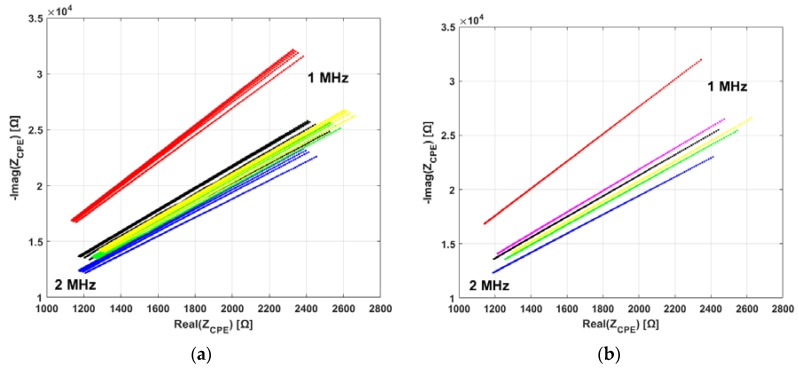
Example of EIS measurement data from the FC bloodline samples. Low and high frequencies are indicated with text in the panels. (**a**) Sets of consecutive measurements performed on the samples. Each color represents different measurements on the same sample; (**b**) Mean impedance spectrum for each group of measurements, color-coded as in the previous panel. Magenta line shows the average of all samples.

**Figure 7:**
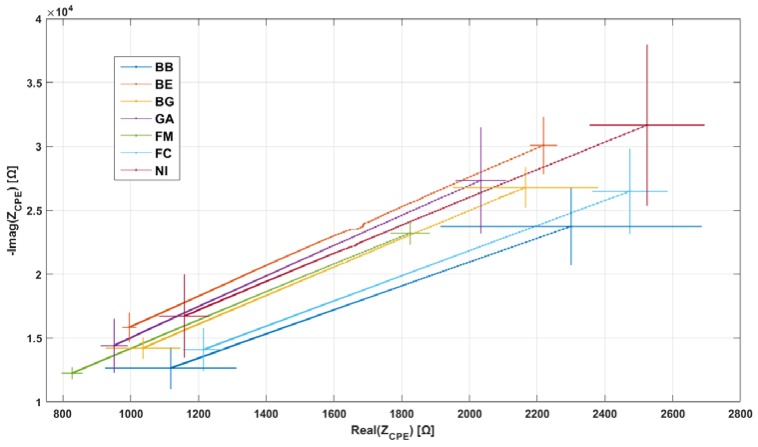
Mean EIS spectra for all bloodline brands.

**Figure 8:**
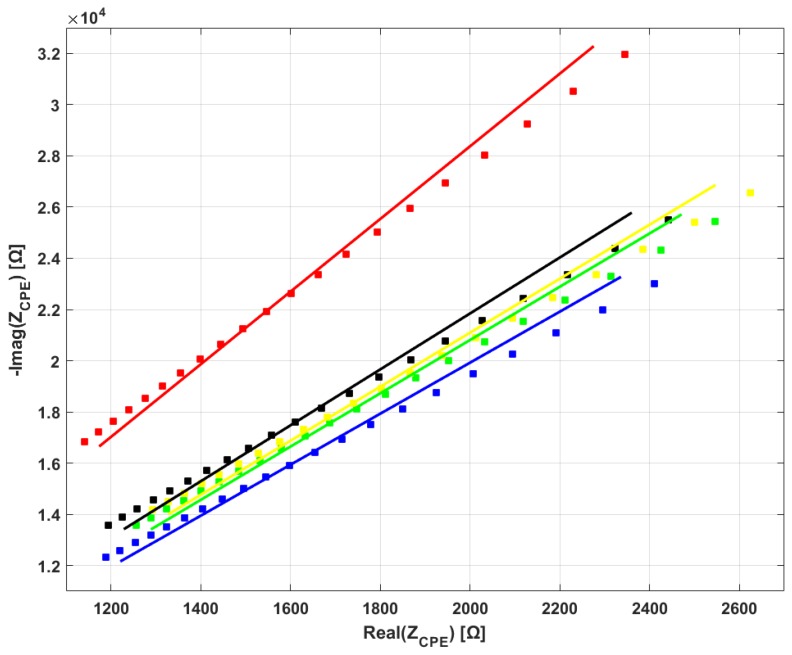
Mathematical fitting of EIS data. Same experimental data (FC bloodline) that was averaged in Figure 7, shown here for the five samples shown in squares. For better illustration, only 1 out of every 10 data points are reported in the figure for each spectroscopy. Colored full lines represent mathematical fitting for each bloodline.

For clarity, in all figures reporting EIS data (Figure 6, Figure 7 and Figure 8), the imaginary part is shown reversed (*y*-axis is inverted). In all figures, the direction of increasing frequency is the one of decreasing impedance. 

Figure 6 shows an example of the results of EIS measurements. Samples from the FC bloodline were chosen as example; however, all bloodlines share similar electrical properties, as shown in Figure 7. Figure 6a shows that all EIS measurements taken on the same sample (same color in panel) are very closely grouped. Variations in measurement results between samples from the same bloodline are most probably the result of slight differences due to the hand-made fabrication of the samples. In Figure 6b, the mean EIS spectra for each sample are shown (same color code as Figure 6a). Figure 6b also shows the final average of the five mean spectra (magenta line) for FC. Similar averages of the EIS results for each bloodline are presented together in Figure 7, for visual comparison. In Figure 7, error bars representing standard deviation between samples are also shown. 

Figure 7 shows that, although there are differences between the impedances of the various brands, all of them manifest CPE behavior. This is evident by the fact that only a constant-phase element shows a decrease of real and imaginary part of impedance with increasing frequency. This electrical behavior is not achievable by any finite combination of resistor and capacitor elements. Moreover, at the chosen frequency interval, impedance is comprised in a similar range for all samples. Figure 8 shows an example of the results of the mathematical fitting of the EIS data with the CPE model. For each of the mean spectra shown in Figure 6b (squares, same color code) the value of Z_CPE_, fitted according to Equation (5), is shown in the same frequency range of 1–2 MHz.

Table 2 reports the numerical results of the mathematical fitting process. The first column shows that all bloodlines have similar values for n at ≃0.94–0.95. However, the standard deviation for each brand value is small, meaning that each group of samples has low variability on this parameter. This is expected, since n is more related to material composition than to sample preparation. In addition, the value of n is close to 1, which would mean perfect capacitive behavior. Nonetheless, the very small value of Q_0_ in the denominator of Equation (5) means that even small deviations of n from unity have a significant CPE effect. The second column reports Q_0_ values. ANOVA testing of parameters Q_0_’ and n showed the presence of statistically significant differences in both cases (*p* = 0.0143 for Q_0_’ and *p* < 0.001 for n). Statistically significant differences between bloodlines are reported in Table 2. 

## 4. Discussion

### 4.1. Analysis of the Results

In this study, the optical and electrical properties of bloodlines for hemodialysis from different manufacturers were estimated and compared. The main goal of the study was to investigate whether significant differences exist that could influence the performance of blood-side contactless sensor systems, a topic for which there is ongoing interest in hemodialysis.

Samples prepared from all bloodlines showed similar behavior regarding optical absorbance: an exponential-like decay starting in the VIS band which reaches flatness in the NIR band, with just a small absorbance peak at 915 nm. Thickness normalization of the samples had the effect of bundling together a few of the spectra, possibly because the involved bloodlines have a very similar chemical composition. This similarity emerges when the effect of differences in thickness is eliminated. However, from visual observation of the results reported in Figure 5b, it is clear that, even after normalization, the spectra present similar shapes but are different quantitatively—for example, if average absorbances or the rates of exponential decay are compared. Only one bloodline with a qualitatively different absorbance spectrum is present in our dataset, as already highlighted in the Results section (samples from the GA brand). As shown in Figure 5, such spectrum presents the basic shape of the spectra from the other brands, however an increase in absorbance is superimposed in the 500–700 nm band. In this band, the most relevant wavelengths for HD are in the 650–700 nm range, which is useful for computation of oxygen saturation (see for example [37] for an application of oxygen saturation monitoring in HD). This spectrum is most likely the result of a different chemical composition of the bloodline. Overall, the results suggest that any kind of absorption-based contactless sensing can be performed on blood across the bloodline’s material, although wavelengths in the 600–1000 nm range will offer lower bloodline absorbance and thus more sensitivity to the properties of blood. The fact that almost all bloodlines have spectra of similar shape and that all have, on average, similar absorbance values implies that optical devices could be used on different bloodlines with only slight recalibrations needed. Specifically, recalibration could involve the use of one single scaling coefficient for the bloodlines that showed very similar spectra, such as the ones closely bundled in Figure 5; on the other hand, recalibration could be wavelength-specific when transitioning to bloodlines with more distinct features.

Regarding electrical impedance, we found that all analyzed bloodlines share the same electrical behavior, that of the constant phase element. To the best of our knowledge, the CPE nature of the bloodlines has only ever been reported in our previous work, in which samples from manufacturer BG underwent electrical analysis in our search to develop a contactless conductivity sensor. In that work, we observed CPE behavior in bloodline samples and whole bloodlines; in this latter case, polymer was mechanically clamped to the electrodes, thus the CPE effect cannot be ascribed to the presence of glue in the samples. The second result of our electrical analysis is of a more quantitative nature and regards statistical analysis of the mathematically-fitted CPE parameters. Analysis of parameter n, an indicator of the “strength” of the CPE effect in the material, revealed that significant differences are present among singles bloodlines and groups. Analysis of the thickness-normalized “modulus” parameter Q_0_’ showed the same result. The consequence of these results is that the overall impedance of the bloodline will differ among distinct brands; however, the estimated ranges of variations in Q_0_’ and n are narrow enough that the impedance modification will not be excessive (i.e., it will not change by an order of magnitude). 

Overall, these results are relevant for the development of contactless electrical sensors like the one created by our group [27]. Moreover, while being outside the body due to extracorporeal circulation, blood might be subject to more electromagnetic power absorption than normal. Thus, knowledge of the EM properties of bloodlines may be used to evaluate how much electrical power is coupled into the flowing blood by external power sources.

### 4.2. Limitations of the Study and Future Directions

One of the limitations of this study is that, although the electrical and optical analyses were performed on multiple material samples, all samples were prepared from a single bloodline kit for each brand. Thus, the study considers some measure of variability in the measurement and sample preparation processes, but it does not investigate variability due to the bloodline manufacturing process. However, bloodline manufacturers usually have extremely strict quality control procedures, so variability in composition and physical properties between bloodlines should be minimal. Thus, results obtained in this study should hold significance independently of this limitation. 

The manufacturing of samples for electrical measurements was manual. However, we feel that the number of samples averaged for the estimation of the electrical properties was sufficiently high to account for the manufacturing differences between the samples related to manual operations.

Another aspect of the study, which may be considered a limitation, is the choice of measurement intervals for optical wavelengths and electrical excitation frequencies. For optical measurements, the VIS-NIR (visible and near-infrared) range was chosen, excluding UV (ultraviolet) light and higher IR wavelengths (middle and far infrared). However, this choice has its justifications:

(1) UV light is damaging for biological tissues, so it cannot be realistically considered for continuous measurements on blood that will be reinjected into the organism.
(2) Optical absorbance of blood at higher infrared wavelengths is dominated by water absorption.

Thus, analyzing bloodline properties beyond the chosen range of the optical spectrum would not lead to information useful for sensing applications—unless optical sensing techniques different from absorbance measurements are considered, which may be an object of future investigation. 

The range of EIS measurements is based on the properties of the analyzed polymers and the limits of the measurement system (as explained in more detail in Section 2). Furthermore, using EIS as a sensing technique at higher frequencies would increase the risk of interference and stray capacitive effects, thus we believe the current range is appropriate.

Future directions to expand on the work in this study include:

- Repeating our investigation on a larger dataset (more bloodlines for each brand, more samples from each bloodline). In addition, performing sample manufacturing in an automated fashion where possible.
- Performing chemical structure analyses (IR, NMR, elemental analysis, etc.) and other material science analyses (e.g., FTIR) to compare the material composition of the bloodlines. Checking databases of physical properties for polymers where available.
- Investigating additional physical properties of the bloodlines, for example:
  ○ Mechanical properties. These properties are useful to manage the flow of blood along the circuit and the interaction between the machine and the bloodline. They are studied independently by bloodline manufacturers but there are no comparisons in literature.
  ○ Acoustic properties. This may be useful for ultrasound sensing, which is used in HD for bubble detection and to measure total protein concentration in blood.
  ○ Optical index of refraction. This may be useful to estimate the amount of light emitted by optical sensors which is lost by refraction index mismatch across the air–polymer–blood optical path.
  ○ Optical scattering. This property causes photons to deviate from their trajectory. Its estimation may help in determining an additional source of optical power loss in bloodlines.

### 4.3. Conclusions

This study presents important data for those scientists and engineers involved in contactless sensor design for hemodialysis. A comparison of the optical and electrical properties of several bloodlines from different manufacturers has not, to our knowledge, been performed before in an academic context. Our conclusions are the following:

- All bloodlines from the analyzed brands share similar optical spectra in the VIS-NIR range, with one exception of increased absorbance in the VIS region.
- All bloodlines from the analyzed brands share the same electrical behavior, that of the constant-phase element (CPE).
- Significant differences are present in CPE parameters among bloodlines.
- ontactless devices developed for use on one specific bloodline could be used with bloodlines of different manufacturers with proper recalibration.

## Figures and Tables

**Table 1:** List of bloodlines with identification data, abbreviation, internal diameter and thickness.

Manufacturer	Model Name	Composition Information (from Package)	Abbreviation	Internal Diameter (mm)	Thickness (mm)
B.Braun Avitum AG (Germany)	A/V Set	DEHP-FREE PVC	BB	4.83	1.00
Bellco (Italy), now part of Medtronic (USA)	Extracorporeal Bloodlines	PVC V 326-1/F	BE	4.37	1.20
Gambro Dasco S.p.a (Italy), now part of Baxter (USA)	ArtiSet	DEHP-FREE	BG	4.27	1.17
Fresenius Medical Care (Germany)	LifeLine Beta AV-Set ONLINEplus BVM 5008-R	-	FC	4.1	1.17
EffeEmme Fabbricazioni Medicali (Italy)	DiaLine	DEHP-FREE	FM	4.9	1
GAMA Group (Czech Republic)	Standardline DIS 06-16 UNIV	DEHP-FREE (pump segment)	GA	4.5	1.17
NIPRO Corporation (Japan)	NIPRO Set	DEHP-FREE	NI	4.33	1.13

**Table 2:** Results of mathematical fitting of EIS data to the CPE model for each brand.

Brand	N (-) (Mean ± std)	Q_0_ (pS) (Mean ± std)	Q_0_’ (pS∙mm) (Mean ± std)
BB	0.942 ± 0.004 ^(b,d,e,f)^	16.64 ± 1.65	16.64 ± 1.65
BE	0.957 ± 0.003 ^(a,c)^	10.35 ± 1.12	12.42 ± 1.34 ^(c)^
BG	0.951 ± 0.004	12.58 ± 1.04	14.67 ± 1.16
FC	0.942 ± 0.007 ^(b,d,e)^	14.95 ± 3.11	17.50 ± 3.64 ^(b,f)^
FM	0.954 ± 0.002 ^(a,c)^	13.94 ± 0.83	13.94 ± 0.83
GA	0.955 ± 0.007 ^(a,c)^	12.03 ± 2.86	14.08 ± 3.35
NI	0.952 ± 0.007 ^(a)^	10.98 ± 2.72	12.41 ± 3.07 ^(c)^

^(a)^ Different from BB; ^(b)^ different from BE; ^(c)^ different from FC; ^(e)^ different from GA; ^(f)^ different from NI. All differences are intended with *p* < 0.05.

## Supplementary Materials

Original data of this study is available online at http://www.mdpi.com/1996-1944/11/3/438/s1.
