# Supplementary material for: Optical and Electrical Characterization of Biocompatible Polymeric Lines for Hemodialysis Applications
Source: Materials (Basel). 2018 Mar 16;11(3):438. doi: 10.3390/ma11030438 (PMC5873017; doi:10.3390/ma11030438)
Supplement: Supplementary file 1 [file materials-11-00438-s001.zip › Data_for upload/EIS/Instructions.docx]

**Raw_Data**

This folder contains data collected from the LCR-meter.

Naming is: *Bloodline_sampleNumber_measurementNumber*

Example: Baxter-Gambro bloodline, second sample, first measurement: *BG_2_1.csv*

Data is in CSV format (Comma-separated values).

First column and second column are real and imaginary part of impedance (R,X)

Each spectrum is sampled at 201 points between 1 and 2 MHz, linearly spaced (1 MhH, 1.005 MHz, etc. )

**CPE_model_fit_results.xlsx**

This file contains results from fitting EIS data for each sample with CPE model on ZView, according to the equation reported on the article.
